# Supplementary material for: Natural-gradient learning for spiking neurons
Source: eLife. 2022 Apr 25;11:e66526. doi: 10.7554/eLife.66526 (PMC9038192; doi:10.7554/eLife.66526)
Supplement: Supplementary file 1 [file elife-66526-transrepform1.docx]

undefined
